# Construction Notes

**Published:** 1894-02-17

**Authors:** 


### CONSTRUCTION NOTES.

### New Fever Hospital at Stockton.

The new fever hospital contains an area of seven acres, and stands in an open situation 61 feet above ordnance datum. The hospital is designed on the pavilion system, and consists of the following departments: Administrative block, typhoid pavilion, scarlatina pavilion, isolation block, disinfector, wash-house, laundry, and mortuary. In the administrative department there are provided on the ground floor matron's room, doctor's room and dispensary, stores, matron's linen-room, nurses' dining-room, kitchen, scullery, &c.; on the first floor there are 13 bed-rooms, baths, lavatories, &c. The fever pavilions are one storey in height, and each contains two large wards, with accommodation in each for eight to 12 beds In the centre of the building is the nurses' duty-room, overlooking each ward. The floors of the wards are waxed and polished. The vitiated air is extracted by means of specially-constructed zinc ducts, surmounted by Shoreland's patent air pump ventilators. The isolation pavilion is mainly for the purpose of accommodating cases which require identification. There are two wards in this pavilion, which is built on the same system as the others, with nurses' duty-room, &c. The washing laundry, disinfecting block, and mortuary are erected in a line off the isolation block, parallel to it and to the administrative block. A Washington Lyons patent disinfector has been chosen for the disinfecting chamber. The drainage is carefully designed and carried out. The whole of the plans have been prepared by, and the work carried out under the supervision of the borough engineer, Mr. L. F. Campbell, C.E., the building section being carried out by Messrs. J.

Cooke and Sons, of Stockton. The total cost of the erection, exclusive of furnishing, is £8,600.

**The Torbay Hospital, Reconstructed.**

Quite an imposing architectural feature in the town is the new home of the Torbay Hospital: for so thorough has been the process of reconstruction, and so completely is it equipped with all modern scientific appliances, that the old is practically a new building. The alterations show on the basement a kitchen, servants' sitting-rooms, scullery, and other offices. Here also is the dispensary, a consulting-room, a waiting-room, and secretary's office. On the ground floor one of the principal apartments is the board-room. In the opposite wing there is a lofty, well-lighted, and well-ventilated ward, and intermediately are the senior and junior house surgeons' sitting and bed rooms, a casualty room, matron's store, &c. Upon the first floor are two large wards, each intended to receive twelve beds, and there are nurses' sitting and bed rooms. The second floor is differently treated. There is a large ordinary ward, known as "The Florence Nightingale Ward," at one end, but in the other wing there are two wards, one for six patients, and the second, which is intended for an isolation ward, is well ventilated and cut off from other portions of the building. On this floor is the operating-room. The third floor is entirely devoted to nurses and servants' rooms. Running from the top to the bottom of the building is a galvanised iron shaft for dust and dirt, and a zinc shaft for dirty linen, which goes direct down to the laundry. There are two lifts, supplied by Messrs. Waygood and Co., of London. The architects for this most thorough and efficient reconstruction being Messrs. Habersohn and Fawckner, of London.

**Hospital for Sick Poor at Dundee.**

This hospital has been erected to meet a much needed want in Dundee of some suitable accommodation for the sick poor, whose numbers had for some time back overflowed the wards of the poorhouses. On September 12th, 1891, the foundation-stone of this erection was laid with full masonic honours by Sir Archibald Campbell; twenty-seven months from that date the hospital stands complete. The estimated cost of the buildings was £25,000, including furnishing, a sum which is not expected to be exceeded. The plans show the buildings as placed on a commanding site. It is constructed on the pavilion system, providing accommodation for the resident officer, nurses, and servants. These buildings, which are plain but substantial, are divided into three, the administrative block in the centre with two pavilions on either side, two for males and two for females, communication being obtained to each wing by means of a large airy corridor which runs right across the building. The principal wards for the patients are eight in number, and each contains 30 beds—15 on either side. Heating is effected by two Manchester stoves near the centre of the ward. Connected with each of the main wards are smaller apartments capable of accommodating three patients, to be used for cases requiring isolation. The infectious diseases hospital is isolated from the main building. The ground floors will be devoted almost exclusively to skin diseases, and here there are four wards, two for males and two for females. Above, such cases as whooping cough, measles, and ophthalmia will be treated, while a large room is set apart for female cancer patients. All the wards are large and airy, and the building is furnished with kitchen and scullery and all necessary lavatory accommodation. The washing house, a large apartment, is equipped with all the necessaries for the rapid execution of work which will have to be undertaken there. Amongst the apparatus identified with the cleansing department is one of Washington Lyons' steam disinfectors, which occupies a building by itself. An important feature of the hospital arrangements is the fact that it is equipped with a complete system of telephones. The architect designing this hospital was Mr. William Alexander, the contractors for the mason work being Messrs. A. and P. Craig.

